# Deep Tumor Penetration of Doxorubicin-Loaded Glycol Chitosan Nanoparticles Using High-Intensity Focused Ultrasound

**DOI:** 10.3390/pharmaceutics12100974

**Published:** 2020-10-15

**Authors:** Yongwhan Choi, Hyounkoo Han, Sangmin Jeon, Hong Yeol Yoon, Hyuncheol Kim, Ick Chan Kwon, Kwangmeyung Kim

**Affiliations:** 1 KU-KIST Graduate School of Converging Science and Technology, Korea University, 145 Anam-ro, Seongbuk-gu, Seoul 02841, Korea; 113377@kist.re.kr; 2 Center for Theragnosis, Biomedical Research Institute, Korea Institute of Science and Technology (KIST), Seoul 02792, Korea; zingouki@gmail.com (H.H.); jeon@kist.re.kr (S.J.); seerou@kist.re.kr (H.Y.Y.); 3 Department of Chemical & Biomolecular Engineering, Sogang University, Shinsu-dong, Mapo-gu, Seoul 121-742, Korea; hyuncheol@sogang.ac.kr

**Keywords:** glycol chitosan nanoparticle, high-intensity focused ultrasound, deep tumor penetration, dense ECM, cancer treatment

## Abstract

The dense extracellular matrix (ECM) in heterogeneous tumor tissues can prevent the deep tumor penetration of drug-loaded nanoparticles, resulting in a limited therapeutic efficacy in cancer treatment. Herein, we suggest that the deep tumor penetration of doxorubicin (DOX)-loaded glycol chitosan nanoparticles (CNPs) can be improved using high-intensity focused ultrasound (HIFU) technology. Firstly, we prepared amphiphilic glycol chitosan-5β-cholanic acid conjugates that can self-assemble to form stable nanoparticles with an average of 283.7 ± 5.3 nm. Next, the anticancer drug DOX was simply loaded into the CNPs via a dialysis method. DOX-loaded CNPs (DOX-CNPs) had stable nanoparticle structures with an average size of 265.9 ± 35.5 nm in aqueous condition. In cultured cells, HIFU-treated DOX-CNPs showed rapid drug release and enhanced cellular uptake in A549 cells, resulting in increased cytotoxicity, compared to untreated DOX-CNPs. In ECM-rich A549 tumor-bearing mice, the tumor-targeting efficacy of intravenously injected DOX-CNPs with HIFU treatment was 1.84 times higher than that of untreated DOX-CNPs. Furthermore, the deep tumor penetration of HIFU-treated DOX-CNPs was clearly observed at targeted tumor tissues, due to the destruction of the ECM structure via HIFU treatment. Finally, HIFU-treated DOX-CNPs greatly increased the therapeutic efficacy at ECM-rich A549 tumor-bearing mice, compared to free DOX and untreated DOX-CNPs. This deep penetration of drug-loaded nanoparticles via HIFU treatment is a promising strategy to treat heterogeneous tumors with dense ECM structures.

## 1. Introduction

Anticancer drug-loaded nanoparticles have been used extensively in cancer treatment. This is because drug-loaded nanoparticles can be efficiently localized at targeted tumor tissues via nanoparticle-derived enhanced permeation and retention (EPR) effects in many pre-clinical tests [1,2,3]. The rapid growth of tumor tissues can cause a leaky vasculature as well as a suppression of lymphatic drainage, resulting in making different characteristics from those of the normal vasculature. In particular, since nanoparticles can extravasate into tumor tissues efficiently via the EPR effect, the EPR effect is regarded as the golden standard in designing nanoparticles for drug delivery [4,5]. Therefore, various nanosized materials, such as liposomes, polymeric nanoparticles, metal nanoparticles, and inorganic nanoparticles have been used in tumor-targeting delivery systems [6,7,8,9]. However, challenges remain to further improve the therapeutic efficacy of drug-loaded nanoparticles in heterogeneous tumors [10,11,12]. In particular, the delivery efficacy of drug-loaded nanoparticles is hampered greatly by limited deep tumor penetration in the complex tumor microenvironment [13,14,15]. It has been known that heterogeneous tumors differ in their vascular structure and perfusion rate [16,17]. Moreover, the thick extracellular matrix (ECM) which consists of collagen and hyaluronan (HA) in the tumor tissue can inhibit the deep tissue penetration of drug-loaded nanoparticles [18,19]. This is because the dense ECM can act as a physical barrier to the accumulation and deep tissue penetration of drug-loaded nanoparticles [15,20]. Thus, the development of a way to ensure a deep penetration of drug-loaded nanoparticles into tumor tissue is an essential challenge to improve the therapeutic efficacy of nanoparticle-based drug delivery systems in cancer treatment.

Many researchers have tried to improve the delivery efficiency of drug-loaded nanoparticles through the remodeling of the ECM in the tumor microenvironment [21,22]. Enzyme-conjugated nanoparticles have been used to increase the deep tissue penetration of nanoparticles through deconstructing the ECM structure, resulting in improvements in the therapeutic efficacy of the tumor. For example, matrix metalloprotease (MMP)-conjugated nanoparticles could break down the ECM structure, resulting in an improved delivery efficiency into deep tumor tissue and the therapeutic efficacy of drug-loaded nanoparticles [23]. Nevertheless, the applications of enzyme-conjugated nanoparticles is still limited due to the complex chemical reactions that bind the enzyme to the nanoparticle surface [24,25]. More practically, high-intensity focused ultrasound (HIFU) technology has been used to break down physically the dense ECM structure in the tumor microenvironment without any toxicity in normal organs. HIFU-mediated drug delivery systems could improve the delivery of high-molecular-weight antibodies and nanoparticles to tumor tissue, due to the successful destruction of the ECM barrier in tumor tissues [26,27]. In our previous report, we reported the exact mechanism of the HIFU-mediated deep tumor penetration of nanoparticles in heterogeneous tumor models [28,29]. Many human solid tumors express high levels of collagen and hyaluronan matrixes that can acts as physical barriers for inhibiting the deep tumor penetration of antibodies and high molecular anticancer drugs [30]. In particular, these EMC-rich tumor tissues composed of highly expressed collagen and hyaluronan matrixes can affect the accessibility and deep tumor penetration of nanosized drug delivery systems in pre-clinical tests [31]. Interestingly, the dense ECM structure of tumor tissues was successfully destroyed by non-invasive pulsed-HIFU exposure. Furthermore, the interstitial flow pressure (IFP) in the tumor tissue was reduced by normalizing the tumor vessels in ECM-rich tumors. Surprisingly, intravenously injected nanosized nanoparticles could be successfully accumulated at in ECM-rich tumors exposed to non-invasive HIFU treatments. These overall results demonstrate that ECM remodeling by HIFU treatment is a promising strategy to enhance the deep tumor penetration and enhanced tumor targeting of drug-loaded nanoparticles in solid tumors.

Glycol chitosan is a natural polysaccharide which is derived from chitosan, it has biocompatibility, non-toxicity, biodegradability, and easy fabrication properties [32]. Notably, a large number of reactive functional groups (primary amine and hydroxyl group) on the glycol chitosan backbone can be modified with cholanic acids, hydrotropic oligomer, photosensitizers, and fullerene, resulting in the formulation of nanomedicines for chemotherapy, gene therapy, and photodynamic therapy [33]. Among them, hydrophobically modified glycol chitosan can form self-assembled nanoparticles due to its amphiphilic structure. In particular, the hydrophobic inner cores of glycol chitosan nanoparticles can be used to deliver theranostic agents such as paclitaxel, docetaxel, and iron oxide nanoparticles via the EPR effect in tumor tissue, resulting in an improved drug delivery efficiency as well as tumor-specific imaging in pre-clinical mice tumor models [34,35,36].

**Figure 1:**
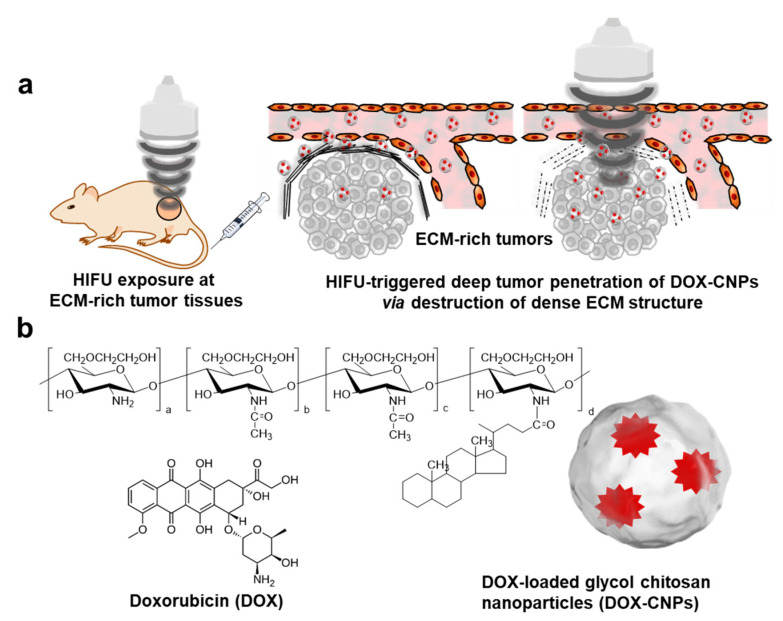
(**a**) High-intensity focused ultrasound (HIFU) treatment of doxorubicin (DOX)-loaded glycol chitosan nanoparticles (CNPs) (DOX-CNPs) to increase their deep tumor penetration in extracellular matrix (ECM)-rich tumor models. (**b**) Schematic illustration of glycol chitosan-5β-cholanic acid conjugate. The glycol chitosan-5β-cholanic acid conjugates form self-assembled nanoparticles in aqueous condition. The anticancer drug of DOX can be loaded into CNPs via a dialysis method, resulting in DOX-CNPs.

Herein, we evaluate the drug delivery efficacy and therapeutic efficacy of HIFU-triggered drug-loaded nanoparticles at ECM-rich tumor models, wherein the ECM-rich tumor tissues were treated with HIFU to destroy the dense ECM structure at A549 tumor tissues (Figure 1a). First, we prepared doxorubicin-loaded glycol chitosan nanoparticles as model drug-loaded nanoparticles. We expect that doxorubicin (DOX)-chitosan nanoparticles (CNPs) are very suitable as model drug delivery systems, due to their high-tumor-targeting ability and low systemic toxicity in vivo [37]. The biodegradable and hydrophilic glycol chitosan polymers were modified hydrophobic 5β-cholanic acid and the conjugates were self-assembled to form glycol chitosan nanoparticles (CNPs). Next, the anticancer drug doxorubicin (DOX) was loaded into CNPs via a simple dialysis method, resulting in DOX-loaded CNPs (DOX-CNPs). The in vitro drug release, cellular uptake and cytotoxicity of HIFU-triggered DOX-CNPs were characterized in cultured cells. Finally, the deep tumor penetration and therapeutic efficacy of HIFU-triggered DOX-CNPs were carefully examined in an ECM-rich A549 tumor animal model, compared to free DOX and untreated DOX-CNPs.

## 2. Materials and Methods

### 2.1. Materials

Glycol chitosan (MW = 250 kDa; degree of deacetylation > 60%), doxorubicin hydrochloride (DOX-HCl), 5β-cholanic acid, 1-ethyl-3-(3-dimethylaminopropyl)-carbodiimide hydrochloride (EDC), N-hydroxysuccinimide (NHS), triethylamine (TEA), anhydrous methanol and anhydrous dimethyl sulfoxide (DMSO) were purchased from Sigma-Aldrich (Merck, Darmstadt, Germany). A fluorescent molecule, Cy5.5-NHS ester, was purchased from Lumiprobe Corporation (Hunt Valley, MD, USA). All other chemicals were purchased as reagent grade and used without further purification or modification.

### 2.2. Synthesis and Characterization of Doxorubicin-Loaded Glycol Chitosan Nanoparticles (DOX-CNPs)

To prepare glycol chitosan nanoparticles (CNPs), hydrophobic 5β-cholanic acid was chemically conjugated to the hydrophilic glycol chitosan through amide linkage formation in the presence of EDC and NHS [15,38]. Briefly, 150 mg of 5β-cholanic acid was dissolved in 120 mL of methanol and mixed with EDC (120 mg) and NHS (72 mg). A total of 500 mg of glycol chitosan was dissolved in 120 mL of methanol/deionized distilled water solution (1:1 *v/v*), followed by slow mixing with 5β-cholanic acid solution. The mixture was vigorously stirred at room temperature for 24 h, and then purified by dialysis against distilled water/methanol (1:1 and 1:0 *v/v*) using a Spectra/Por^®^4 dialysis membrane (MWCO = 12–14 kDa, Repligen Corporation, Waltham, MA, USA). The resulting solution was lyophilized to obtain a white powder of CNPs. For the in vitro and in vivo fluorescence monitoring of CNPs, CNPs were chemically modified with near-infrared fluorescence (NIRF) dye, Cy5.5. In brief, 100 mg of CNPs were dissolved in 40 mL of DMSO, followed by mixing with 1 mL of DMSO containing 1 mg of Cy5.5-NHS. The mixture was stirred for 12 h at room temperature. Subsequently, the mixture was purified by dialysis against distilled water for 2 days using a Spectra/Por^®^ 4 dialysis membrane (MWCO = 12–14 kDa, Repligen Corporation, Waltham, MA, USA). The resulting solution was lyophilized to obtain Cy5.5-conjugated CNPs.

To prepare DOX-encapsulated CNPs (DOX-CNPs), DOX-HCl was physically encapsulated into CNPs using a simple dialysis method. In brief, 50 mg of CNPs was dissolved in 10 mL of DMSO/distilled water (1:1 *v/v*). A total of 21.4 mg of DOX-HCl was dissolved in 2 mL of DMSO/distilled water (1:1 *v/v*). Then, the DOX-HCl solution was treated 10.8 μL of TEA for desalting, followed by mixing with CNP solution. The mixture was purified by a dialysis against distilled water for 12 h using a Spectra/Por^®^ 4 dialysis membrane (MWCO = 12–14 kDa, Repligen Corporation, Waltham, MA, USA). The resulting solution was filtered using 0.8 μm syringe filter, followed by lyophilizing to obtain DOX-CNPs.

To confirm the hydrodynamic diameter of CNPs and DOX-CNPs, 1 mg of CNPs and DOX-CNPs were dispersed into 1 mL of PBS (pH 7.4) using a probe-type sonicator (Amp 21%, 1 min, VCX-750, Sonics and Materials, Newtown, CT, USA). The volume-weighted size distribution and zeta potential of CNPs and DOX-CNPs were measured using dynamic light scattering (DLS, Nano ZS, Malvern Panalytical Ltd., Grovewood Road, Malvern, UK) at 25 °C. The size stability and volume-weighted size distribution of CNPs and DOX-CNPs were monitored in both PBS (pH 7.4) and 1% FBS-containing PBS (pH 7.4) conditions using DLS. The morphologies of CNPs and DOX-CNPs were observed using transmission electron microscopy (TEM, CM30 Electron Microscope, Philips, CA, USA) at 200 eKV of an accelerating voltage. For TEM images, CNPs and DOX-CNPs were dispersed in distilled water and 5 μL of CNPs or DOX-CNPs solution was dropped on a 200 mesh carbon-coated copper grid, followed by negative staining using 2% uranyl acetate solution. Based on TEM images (*n* = 10), size distribution of CNPs and DOX-CNPs were determined using Image Pro Plus4.5 5 software (Media Cybernetics, Bethesda, Rockville, MD, USA).

### 2.3. In Vitro Release Profile of HIFU-Triggered DOX-CNPs

An in vitro DOX release from DOX-CNPs was observed in 37 °C PBS (pH 7.4) containing 0.1% of Tween 80. To observe the HIFU-triggered DOX-release, 10 mg of DOX-CNPs was dispersed in PBS (2 mL) and placed in a dialysis membrane (MWCO = 100 kDa, Repligen Corporation, Waltham, MA, USA) (*n* = 3). The membrane was transferred into a 50 mL conical tube filled with 10 mL of PBS (pH 7.4) containing 0.1% of Tween 80. The conical tubes were placed in a 37 °C water bath then shaken horizontally (100 rpm). The dialysis membrane was treated with HIFU in destruction mode for 5 min (power: 10 MHz, mechanical index: 0.235) using a High-Resolution Micro-Imaging System (Vevo 770, Visualsonics, Toronto, Maryland, Canada). The amount of DOX released from CNPs at a pre-determined time point was determined using an absorbance at 490 nm measured by the UV–Vis spectrometer (G1103A, Agilent, Santa Clara, CA, USA).

### 2.4. Cellular Uptake Behavior of HIFU-Triggered DOX-CNPs

Human non-small cell lung tumor cells, A549, was cultured in Roswell Park Memorial Institute (RPMI) 1640 media (Welgene, Daegu, Korea) containing 10% fetal bovine serum (FBS) and 1% penicillin/streptomycin at 37 °C in a 5% CO_2_ incubator. To demonstrate the cellular uptake of DOX-CNPs, A549 cancer cells (1 × 10^4^ cells) were seeded onto 35 mm glass bottom dish and incubated for 24 h. After 24 h post-incubation, the medium was replaced with 1 mL of FBS-free RPMI-containing Cy5.5-labeled DOX-CNPs (1 μg/mL of DOX, 1 mL). To characterize the cellular uptake mechanism of the HIFU-triggered cellular uptake mechanism of DOX-CNPs, DOX-CNP-treated A549 cells were treated with HIFU in destruction mode (power: 10 MHz, mechanical index: 0.235) for 5 min and then incubated for pre-determined time. DOX-CNP-treated A549 cells were washed twice using Dulbecco’s Phosphate Buffered Saline (DPBS) and fixed with a 4% paraformaldehyde solution for 10 min. The nuclei of A549 cells was stained with DAPI for 5 min at room temperature. The fluorescence from A549 cells was visualized using confocal laser scanning microscopy (Leica TCS SP8, Wetzlar, Land Hessen, Germany) equipped with 405-diode (405 nm), Ar (488 nm), and HeNe-Red (633 nm) lasers. Tumor tissue fluorescence images were acquired using LAS X software (Leica Microsystems, Wetzlar, Land Hessen, Germany). The fluorescence of DOX and CNP was measured using Image Pro Plus 4.5 5 image analysis software (Media Cybernetics, Bethesda, Rockville, MD, USA).

### 2.5. In Vitro Cytotoxicity Test of HIFU-Triggered DOX-CNPs

The A549 tumor cells (5 × 10^3^ cells/well) were seeded onto 96-well plates and stabilized for 12 h. The A549 cells were then incubated with various concentrations (0, 0.1, 1, 10, 100 and 500 μg/mL of DOX) of free DOX, CNPs and DOX-CNPs for 24 h. To measure cell viability, 10% (*v/v*) CCK-8 solution was added to each well, followed by further incubation for 1 h at 37 °C. The absorbance at 450 nm was measured using a microplate reader (VERSAmax™, Molecular Devices Corp., Sunnyvale, CA, USA).

To analyze the cell viability after HIFU treatment, A549 cells were incubated with CNPs and DOX-CNPs (100 μg/mL of DOX) for 24 h. These A549 cells were exposed to HFU in destruction mode (power: 10 MHz, mechanical index: 0.235) for 5 min, followed by further incubation for 24 h at 37 °C in a 5% CO_2_ incubator. Finally, the A549 cells were washed twice with DPBS. Subsequently, 10% (*v/v*) CCK-8 solution was added to each well, followed by further incubating for 1 h at 37 °C. The absorbance at 450 nm was measured using a microplate reader (VERSAmax™, Molecular Devices Corp., Sunnyvale, CA, USA).

### 2.6. In Vivo Biodistribution of HIFU-Triggered DOX-CNPs in A549 Tumor-Bearing Mice

All animal experiments were performed in compliance with the guidelines of the Institutional Animal Care and Use Committee (IACUC) in the Research Animal Resource Center of Korea Institute of Science and Technology (approved number: 2017-109). To establish A549 tumor-bearing mice, 1 × 10^7^ cells of A549 cells were inoculated in the left flank of male Balb-c/nude mice (4 weeks old, ORIENT BIO Inc., Gyeonggi-do, Korea). When the tumor volume reached approximately 250 ± 50 mm^3^, Cy5.5-labeled DOX-CNPs (20 mg/kg, 100 µL) was injected through the tail vein. HIFU (VIFU 2000, ALPINION, Gyeonggi-do, Korea) was applied at the tumor site for 5 min simultaneously with an intravenous injection of Cy5.5-DOX-CNPs as pre-set conditions (intensity: 5 W/cm^2^, frequency: 1.5 MHz, duty cycle: 10%, pulse repetition frequency: 1 Hz, time per spot: 30 s, interval: 2 mm). Near infrared fluorescence (NIRF) images of mice animal models were carried out through IVIS SPECTRUM (Xenogen, Alameda, CA, USA). To compare the tumor and organ distributions of Cy5.5-labeled DOX-CNPs, the mice were sacrificed 24 h post-injection. Tumor, liver, lung, spleen, kidney, and heart were dissected from mice, and then NIRF images were obtained via IVIS SPECTRUM.

To observe the deep tissue penetration of Cy5.5-labeled DOX-CNPs, tumor tissues were excised 24 h post-injection of Cy5.5-labeled DOX-CNPs. Excised tumor tissues were embedded in an optimum cutting temperature tissue compound (OCT compound, Sakura Finetek, Chuo-ku, Tokyo, Japan), followed by a transfer to a refrigerator at under −20 °C for 24 h. The tumor tissue blocks were sectioned with a 10 µm thickness with a cryostat (Leica, Bannockburn, IL, USA). The tumor tissue slides were washed with distilled water twice to remove the OCT compound, followed by nuclei staining using DAPI solution for about 10 min. After being washed three times with DPBS, tumor tissue slides were fixed using mounting solution (Vectashield, Vector Laboratories Inc., Burlingame, CA, USA). The fluorescence of the tumor tissue was observed using a fluorescence microscopy (OLYMPUS, Tokyo, Japan).

### 2.7. Antitumor Efficacy of DOX-CNPs with HIFU Treatment

To evaluate antitumor efficacy in animal models, 1 × 10^7^ cells of A549 cells were inoculated in the left flank of male Balb-c/nude mice (4 weeks old, ORIENT BIO Inc., Gyeonggi-do, Korea). When the tumor volume reached approximately 60 ± 5 mm^3^, saline, DOX (2 mg/kg), DOX-CNPs (2 mg/kg of DOX) were injected into the A549 tumor-bearing mice through the tail vein. At 1, 3, 5 and 7 days post-injection, the tumor tissues were treated with HIFU for 5 min as pre-set conditions (intensity: 5 W/cm^2^, frequency: 1.5 MHz, duty cycle: 10%, pulse repetition frequency: 1 Hz, time per spot: 30 s, interval: 2 mm). Tumor volume and survival rate were monitored for 22 days to evaluate the antitumor efficacy of each group.

### 2.8. Histological Analysis

To observe the ECM-rich structure of A549 tumor tissue, collagen in murine squamous cell carcinoma (SCC7) and A549 tumor tissues were stained using Masson’s trichrome staining method [28].

In brief, 1 × 10^6^ cells of SCC7 cells and 1 × 10^7^ cells of A549 cells were inoculated in the left flank of male Balb-c/nude mice (4 weeks old, ORIENT BIO Inc., Gyeonggi-do, Korea). When SCC7 and A549 tumor tissues grew to 250 ± 50 mm^3^, both tumors were excised and fixed in 4% formaldehyde solution. The tumor tissues were then embedded in paraffin after dehydration. Paraffin-embedded tumor tissues were cut to 6 μm thick and tissue slides were stained with Masson’s trichrome staining solution.

Furthermore, ECM structure changes of A549 tumor tissues after HIFU treatment were observed using Masson’s trichrome staining method. In brief, 1 × 10^7^ cells of A549 cells were inoculated in the left flank of male Balb-c/nude mice (4 weeks old, ORIENT BIO Inc., Gyeonggi-do, Korea). When A549 tumor tissues grew to 250 ± 50 mm^3^, the tumor tissues were treated with HIFU for 5 min as pre-set conditions (intensity: 5 W/cm^2^, frequency: 1.5 MHz, duty cycle: 10%, pulse repetition frequency: 1 Hz, time per spot: 30 s, interval: 2 mm). The tumor tissues were then excised and embedded in paraffin after dehydration. Paraffin-embedded tumor tissues were cut to 6 μm thick and tissue slides were stained with Masson’s trichrome staining solution. Blue-stained collagen in the tumor tissue slides were observed using a light microscope (Olympus, Tokyo, Japan). For the quantification of blue-stained collagen areas, they were measured using Image Pro Plus 4.5 5 image analysis software (Media Cybernetics, Bethesda, Rockville, MD, USA).

To observe the organ toxicity of DOX-CNPs according to HIFU exposure, the liver and kidneys were excised from the A549 tumor-bearing mice at 24 h post-injection of DOX-CNPs with HIFU exposure. The excised liver and kidneys were fixed using 4% formaldehyde solution, followed by embedding in paraffin after dehydration. The paraffin-embedded tissues were cut into 6 μm-thick sections and were stained with hematoxylin and eosin (H&E).

To observe morphological changes in the tumor tissues, tumor tissues were excised from the A549 tumor-bearing mice at 22 days post-treatment. The excised tumor tissues were fixed using 4% formaldehyde solution, followed by embedding in paraffin after dehydration. The paraffin-embedded tissues were cut into 6 μm-thick sections and were stained with hematoxylin and eosin (H&E). All histological analysis images were acquired through a light microscope (Olympus, Tokyo, Japan).

### 2.9. Statistical Analysis 

In this study, the statistical differences between each group were analyzed through a one-way ANOVA in the Origin 2020 software (OriginLab Corporation, Northampton, MA, USA). The significant difference was marked with an asterisk (*) in the figures.

## 3. Results and Discussion

### 3.1. In Vitro Characterization of DOX-Loaded Glycol Chitosan Nanoparticles (DOX-CNPs)

**Figure 2:**
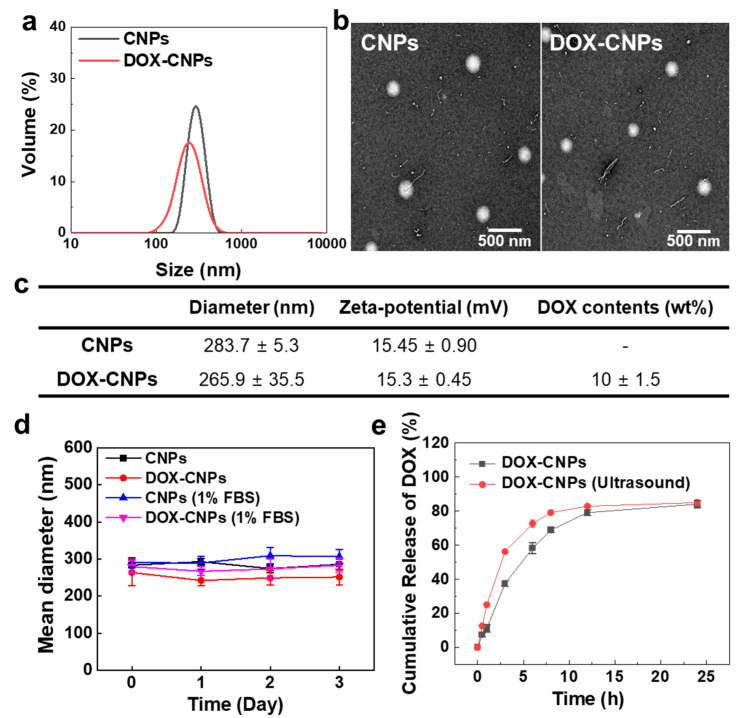
Physicochemical properties of DOX-CNPs in vitro. (**a**) The volume-weighted size distribution of CNPs and DOX-CNPs (1 mg/mL) in PBS (pH 7.4), measured using dynamic light scattering. (**b**) TEM image of CNPs and DOX-CNPs. (**c**) Characteristic table of CNPs and DOX-CNPs summarized diameter, zeta potential, and DOX contents. (**d**) Size stability of CNPs and DOX-CNPs in PBS (pH 7.4) and 1% FBS-containing PBS (pH 7.4) for 3 days. (**e**) In vitro release behavior of DOX from the DOX-CNPs (mean ± SD, *n* = 5). HIFU treatment was carried out for 5 min.

The amphiphilic glycol chitosan-5β-cholanic acid conjugates were prepared by direct coupling between hydrophobic 5β-cholanic acid and hydrophilic glycol chitosan, resulting in the formation of nanoparticles in an aqueous condition (Figure 1b). The glycol chitosan-5β-cholanic acid conjugates contained 162 ± 6.5 molecules of 5β-cholanic acid per glycol chitosan backbone, confirmed by a colloidal titration method [39,40]. For the in vitro and in vivo near-infrared fluorescence (NIRF) imaging, 3.9 molecules of Cy5.5 were chemically conjugated to glycol chitosan-5β-cholanic acid conjugates. Next, the anticancer drug, DOX, was encapsulated into the hydrophobic cores of CNPs via a dialysis method. The amount of DOX in DOX-CNPs was 10 ± 1.5 wt%, calculated by the DOX-standard curve measured at 490 nm in the UV–Vis spectrum. The sizes of CNPs and DOX-CNPs in the aqueous solution measured by DLS were 283.7 ± 5.3 nm and 265.9 ± 35.5 nm. The volume-weighted distribution of CNPs and DOX-CNPs ranged from 260 to 300 nm and 140 to 300 nm, respectively, showing a wider size distribution after DOX encapsulation (Figure 2a). Additionally, the TEM images showed that CNPs and DOX-CNPs had spherical nanoparticle structures with diameters of 262 ± 12 nm and 250 ± 17 nm, respectively (Figure 2b). The size histogram from the TEM images of CNPs and DOX-CNPs showed 200 to 260 nm of distribution, resulting in the similar size distribution of CNPs and DOX-CNPs (Figure S1a and S1b). The surface charges of CNPs and DOX-CNPs were measured to +15.45 ± 0.90 mV and +15.3 ± 0.45 mV, indicating that the surface of CNPs and DOX-CNPs were covered with positively charged glycol chitosan polymers (Figure 2c). Although CNPs and DOX-CNPs had a positive surface, the volume-weighted size distribution of CNPs and DOX-CNPs showed no changes in the size distribution under 1% FBS-containing medium (Figure S1c and S1d). Furthermore, the size of CNPs and DOX-CNPs was stable for 3 days in both PBS (pH 7.4) and 1% FBS-containing PBS (pH 7.4) conditions (Figure 2d). This is because glycol moiety can act as poly(ethylene glycol) (PEG), resulting in the inhibition of size changes by interrupting protein adsorption [41]. This biocompatible glycol chitosan polymer outer surface of CNPs and DOX-CNPs could prevent the adsorption of non-specific proteins in vivo and enables high accumulation at targeted tumor tissues through EPR in vivo [37]. Based on these characterization results, we expect that CNPs can sufficiently encapsulate the anticancer drug DOX into the hydrophobic inner cores of 5β-cholanic acid. Prior to the in vivo study of the DOX-CNPs, in vitro drug release profiles were evaluated. In the case of DOX-CNPs without ultrasound treatment, it was confirmed that the drug was released gradually up to 24 h, but 5 min of HIFU treatment (destruction mode, power: 10 MHz, mechanical index: 0.235) substantially increased the drug release amount by 1.6 and 2.2 times after 30 min or 1 h post-incubation in PBS at 37 °C, compared to untreated DOX-CNPs, due to the ultrasound-triggered rapid drug release from DOX-CNPs (Figure 2e).

### 3.2. In Vitro Cellular Uptake and Cytotoxicity of HIFU-Triggred DOX-CNPs

**Figure 3:**
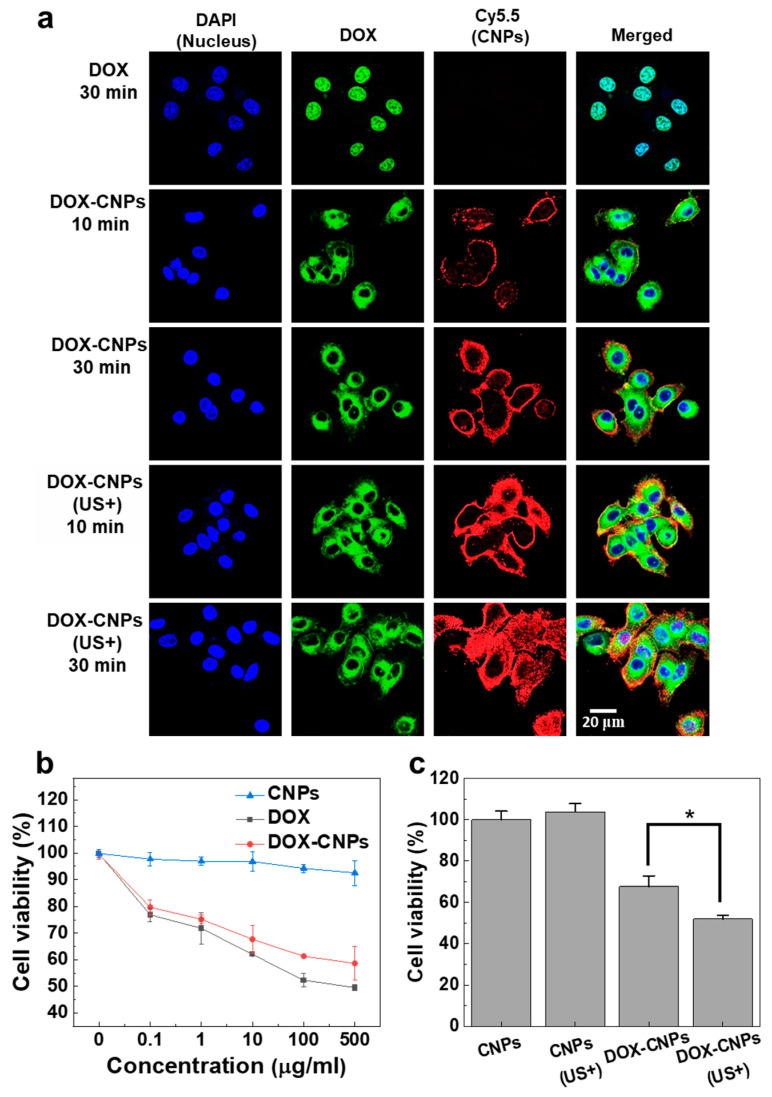
In vitro cellular uptake and cell viability of DOX-CNPs in cultured cells. (**a**) HIFU-triggered (US+) cellular uptake mechanism of DOX-CNPs. A549 cancer cells were incubated with free DOX (1 μg/mL), DOX-CNPs (10 μg/mL) and HIFU-triggered (US+) DOX-CNPs (10 μg/mL) for 10 min and 30 min. DOX-CNP-treated A549 cells were exposed to HIFU in destruction mode (power: 10 MHz, mechanical index: 0.235) for 5 min. (**b**) Cell viability of free DOX, CNPs and DOX-CNPs in cultured A549 cells (mean ± SD, *n* = 5). (**c**) Cell viability of HIFU-triggered DOX-CNPs (10 μg/mL) in A549 cells. CNPs (US+) and DOX-CNPs (US+) groups were treated with HIFU in destruction mode (power: 10 MHz, mechanical index: 0.235) for 5 min and the cell viability was measured 24 h post-incubation (mean ± SD, *n* = 5). (*) indicates difference at the *p* < 0.05 significance level.

To observe the cellular uptake of HIFU-triggered Cy5.5-labeled DOX-CNPs, A549 cells were treated with 100 μg of DOX-CNPs and the cells were exposed to HIFU in destruction mode (power: 10 MHz and mechanical index: 0.235) for 5 min and the cellular uptake of HIFU-triggered Cy5.5-labeled DOX-CNPs were visualized with confocal microscopy (Figure 3a). In the control, without HIFU exposure, Cy5.5-labeled DOX-CNPs (red colors) slowly bound to cell membranes after 10 min post-incubation and then they were internalized into the cytoplasm at up to 30 min, wherein the bright red colors of Cy5.5-labeled DOX-CNPs were clearly observed in the cytoplasm compartment of A549 cells. It is reported that CNPs show a fast uptake into cancer cells via diverse nanoparticle-derived endocytic pathways [42,43,44]. Interestingly, the HIFU-triggered cellular uptake of Cy5.5-labeled DOX-CNPs (red color) was clearly observed after 5 min of HIFU pre-treatment (destruction mode; power: 10 MHz, mechanical index: 0.235), compared to untreated Cy5.5-labeled DOX-CNPs. After 10 min post-incubation, a large amount of Cy5.5-labeled DOX-CNPs in the cell membrane and cytoplasm were observed in A547 cells. Furthermore, most DOX-CNPs were rapidly internalized into the cytoplasm compartment of A549 cells after 30 min post-incubation, due to the HIFU-triggered rapid cellular uptake mechanism. More importantly, the rapid cellular uptake of DOX (green color) in CNPs was clearly observed via the HIFU-triggered fast cellular uptake of DOX-CNPs, compared to free DOX. In the case of HIFU exposure, the cellular uptake of Cy5.5-labeled DOX-CNPs increased by 1.6 and 2.0 times after 10 and 30 min post-incubation, compared to untreated DOX-CNPs (Figure S2). Finally, after 30 min post-incubation, the cellular uptake of DOX molecules in CNPs increased by 5.1 times via the HIFU-triggered cellular uptake of DOX-CNPs, compared to free DOX. This is because HIFU exposure to live cells can increase the permeability of nanoparticles into cell membranes via the sonoporation effect as well as the mechanical effects of ultrasound [45,46,47].

To evaluate the cytotoxicity of DOX-CNPs in A549 tumor cells, the cell viability of A549 cells was assayed using cell counting kit-8 (CCK-8) at various concentrations of free DOX, CNPs, and DOX-CNPs. CNPs did not show severe cytotoxicity at a high concentration (500 µg/mL) in culture media. However, the cell viability of DOX-CNPs-treated A549 cells gradually decreased, due to the release of free DOX from DOX-CNPs (Figure 3b). Furthermore, the cell viability of free DOX-treated A549 cells decreased in a DOX concentration-dependent manner. Interestingly, HIFU exposure could increase the cytotoxicity of DOX-CNPs in cultured cells, compared to untreated DOX-CNPs (Figure 3c). When the A549 cells were treated with 100 μg of DOX-CNPs (10 μg of DOX) for 24 h, the cell viability was measured at 67.68 ± 5.07%. However, after HIFU exposure for 5 min, the cell viability of DOX-CNP-treated A549 decreased to 51.99 ± 1.79%. It is deduced that the rapid cellular uptake and the rapid drug release of HIFU-triggered DOX-CNPs could increase the cytotoxicity of drug-loaded nanoparticles in cultured cells, compared to untreated DOX-CNPs.

### 3.3. In Vivo Biodistribution and Therapeutic Efficacy of HIFU-Triggered DOX-CNPs

The in vivo biodistribution of Cy5.5-labeled DOX-CNPs without or with HIFU treatment was monitored in ECM-rich A549 tumor-bearing mice. This is because A549 tumor tissues with stiff ECMs composed of dense collagen and hyaluronan could prevent the deep tissue penetration of nanosized drug carriers [15,28]. Prior to monitor-targeted tumor accumulation, we firstly confirmed the collagen contents of tumor tissues using A549 and SCC7 tumor-bearing mice. When the tumor volume reached to 250 ± 50 mm^3^, tumor tissues were excised from the mice, followed by staining using Masson’s trichrome staining solution. Compared to SCC7 tumor tissue images, A549 tumor tissue images showed a widely dispersed collagen area which was blue-stained by Masson’s trichrome staining solution (Figure S3a). Furthermore, the blue-colored collagen fibers were intricately connected to each other throughout A549 tumor tissues, indicating ECM-rich tumor tissues. However, in the case of SCC7 tumor tissues, almost no collagen fibers were seen in ECM-less tumor tissues. In particular, the amount of collagen fibers in A549 tumor tissues was eight times higher than that of SCC7 tumor tissues (Figure S3b).

Next, we confirmed a tumor tissue collagen destruction effect by HIFU treatment. The A549 tumor model was established by a subcutaneous injection of 1 × 10^7^ cells into the Balb-c/nude mice. When the tumor was grew up 250 ± 50 mm^3^, A549 tumor tissues were treated with HIFU (intensity: 5 W/cm^2^, frequency: 1.5 MHz, duty cycle: 10%, pulse repetition frequency: 1 Hz, and time per spot: 30 s) for 5 min. Then, A549 tumor tissues were excised from the mice, followed by staining using Masson’s trichrome staining solution. A549 tumor tissue images showed a widely dispersed blue-stained collagen area whereas the blue-stained collagen area was dramatically reduced after HIFU treatment (Figure S4a). Furthermore, the blue-stained collagen area of the HIFU-treated A549 tumor tissue observed was six times lower than that of the A549 tumor tissue, resulting in a significant reduction in collagen in the tumor tissue by HIFU treatment (Figure S4b).

**Figure 4:**
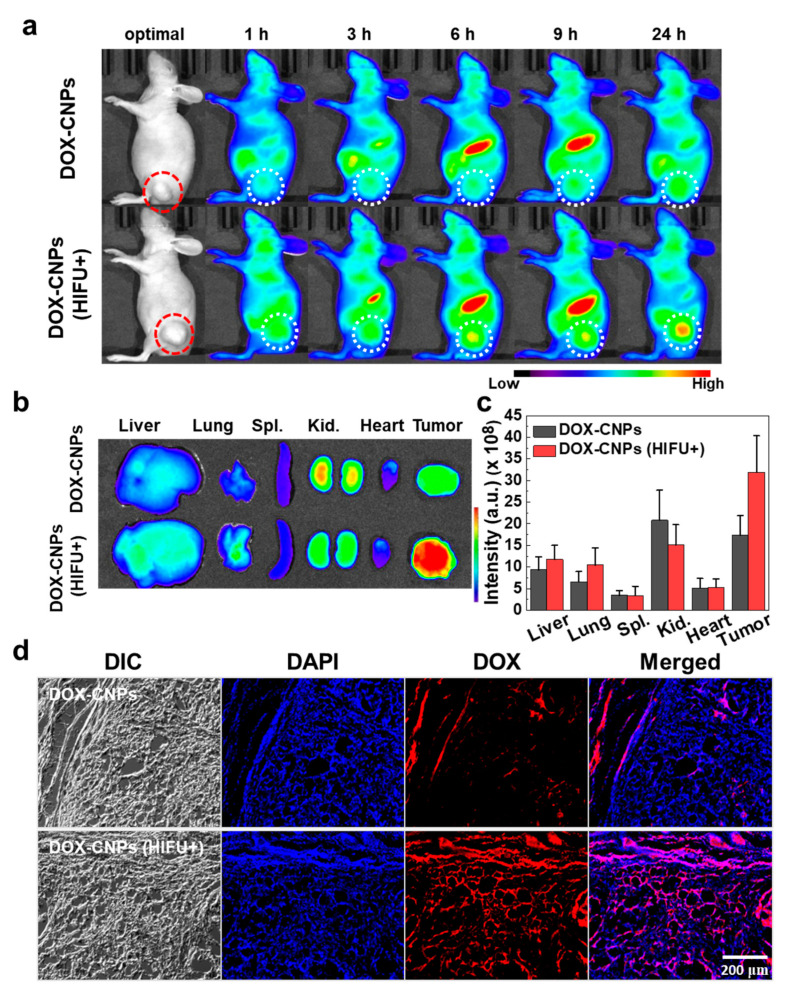
In vivo near-infrared fluorescence (NIRF) imaging of Cy5.5-labeled DOX-CNPs in ECM-rich A549 tumor animal model. (**a**) Biodistribution of Cy5.5-labeled DOX-CNPs without and with HIFU treatment (intensity: 5 W/cm^2^, frequency: 1.5 MHz, duty cycle: 10%, pulse repetition frequency: 1 Hz, time per spot: 30 s, interval: 2 mm, expose time: 5 min). The red and white dot circles indicate tumor site. (**b**) Ex vivo NIRF imaging of liver, lung, spleen, kidney, heart, and tumor at 24 h post-injection. (**c**) Mean NIRF signal intensity of ex vivo NIRF image (Spl.; spleen, Kid.; kidney). (**d**) Ex vivo NIRF microscopic images of deep tumor penetration of untreated Cy5.5-labeled DOX-CNPs and HIFU-treated Cy5.5-labeled DOX-CNPs in ECM-rich tumor tissues.

To monitor the targeted tumor accumulation of DOX-CNPs, the A549 tumor model was made by a subcutaneous injection of 1 × 10^7^ cells into the Balb-c/nude mice. When the tumor volume reached 250 ± 50 mm^3^, Cy5.5-DOX-CNPs (20 μg/kg, 100 μL) were intravenously injected into the A549 tumor-bearing mice (*n* = 3). After an intravenous injection of Cy5.5-DOX-CNPs, the tumor was treated with HIFU (intensity: 5W/cm^2^, frequency: 1.5 MHz, duty cycle: 10%, pulse repetition frequency: 1 Hz, and time per spot: 30 s) for 5 min and the tumor-targeting ability of Cy5.5-DOX-CNPs were visualized using non-invasive NIRF imaging. As we expect, 6 h post-injection, HIFU-treated groups showed a high NIRF intensity of Cy5.5-labeled DOX-CNPs at targeted tumor tissues (white dotted circle), compared to untreated DOX-CNP groups (Figure 4a). The tumor accumulation of HIFU-treated DOX-CNPs groups increased noticeably via the nanoparticle-derived EPR effect for 24 h, whereas untreated DOX-CNP groups slightly accumulated at targeted tumor tissues up to 24 h post-incubation, due to the dense ECM structure of A549 tumor tissues. To observe ex vivo NIRF images, the tumor tissues and the major organs, including liver, lung, spleen, kidney, and heart, were excised from the tumor-bearing mice at 24 h post-injection (Figure 4b). The bright NIRF signal of HIFU-treated DOX-CNPs was clearly observed at targeted tumor tissues. In the control, both DOX-CNPs without and with HIFU treatment showed the similar non-specific accumulations in normal tissues, such as liver, lung, and kidney. In the HIFU-treated groups, the NIRF signal intensity of Cy5.5-labeled DOX-CNPs in the tumor tissue was 1.84 times higher than that of the untreated groups (Figure 4c). It is deduced that HIFU treatment at targeted tumor tissues could destroy dense ECMs composed of collagen and hyaluronan, resulting in the deep tumor penetration of DOX-CNPs at ECM-rich A549 tumor tissues [28,48]. Surprisingly, intravenously injected DOX-CNPs could be successfully accumulated at in ECM-rich tumors exposed to HIFU treatments. Lastly, the excised tumor tissues were further observed using fluorescence microscopy. The NIRF microscopic images showed that untreated DOX-CNPs mainly localized in the boundary region of ECM-rich tumor tissues, indicating that dense ECM structures inhibited the deep tumor penetration of drug-loaded nanoparticles (Figure 4d). However, HIFU-treated DOX-CNPs localized substantially in the deep inner part of ECM-rich tumor tissues via the HIFU-derived destruction of the dense ECM structure. These results indicate that HIFU treatment on ECM-rich tumor tissues helped the deep tumor penetration of DOX-CNPs in vivo.

### 3.4. In Vivo Therapeutic Efficacy Using HIFU-Triggered DOX-CNPs in A549 Tumor-Bearing Mice

**Figure 5:**
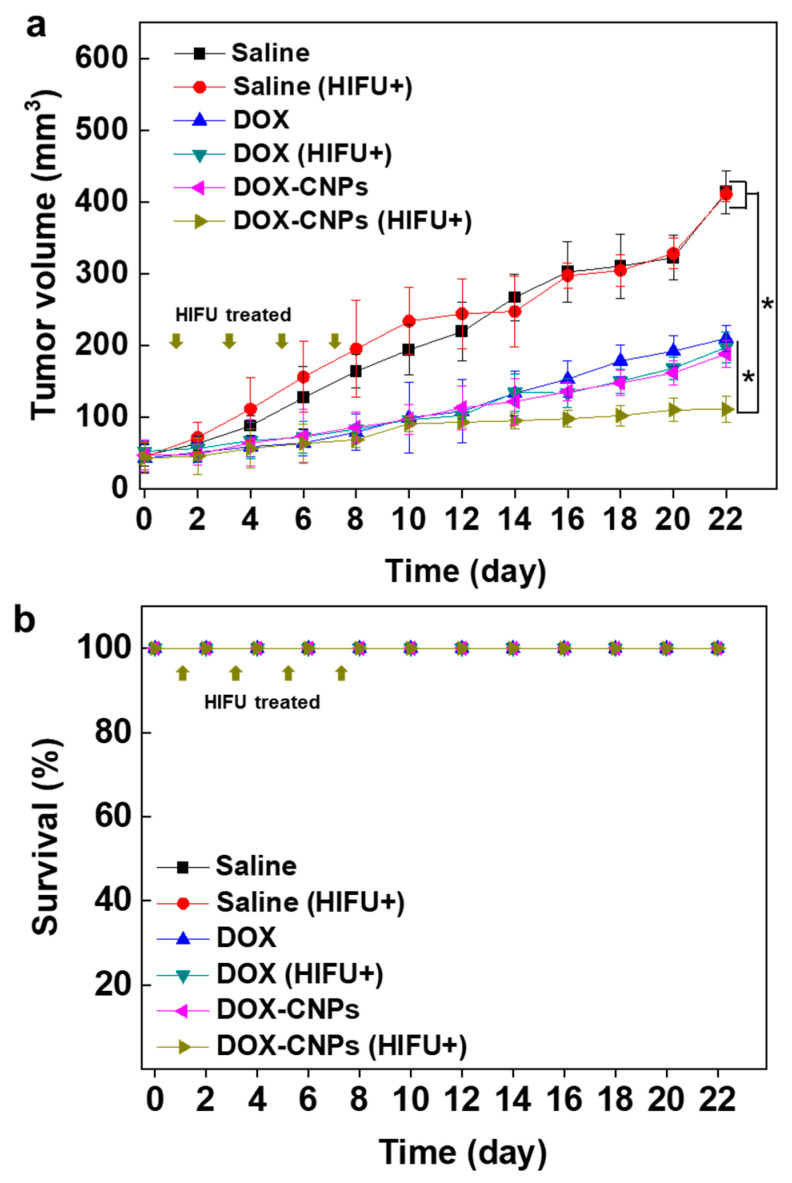
In vivo therapeutic efficacy of HIFU-triggered DOX-CNPs in ECM-rich A549 tumor-bearing mice. (**a**) Antitumor efficacy of DOX-CNPs with HIFU treatment (*n* = 5 per group). The arrows indicate DOX-CNP injection and HIFU treatment (intensity: 5 W/cm^2^, frequency: 1.5 MHz, duty cycle: 10%, pulse repetition frequency: 1 Hz, time per spot: 30 s, interval: 2 mm, expose time: 5 min). (*) indicates difference at the *p* < 0.05 significance level. (**b**) Survival rate of A549 tumor-bearing mice treated with saline, saline (HIFU+), DOX, DOX (HIFU+), DOX-CNPs, and DOX-CNPs (HIFU+). The arrows indicate HIFU treatment.

The in vivo therapeutic efficacy of HIFU-triggered DOX-CNPs in tumors was monitored up to 24 days. When tumors grew to approximately 60 ± 5 mm^3^, saline, DOX (2 mg/kg), DOX-CNPs (2 mg/kg of DOX) were injected into the A549 tumor-bearing mice through the tail vein. At 1, 3, 5 and 7 days after injection, tumor tissues were treated with HIFU (+HIFU) (intensity: 5 W/cm^2^, frequency: 1.5 MHz, duty cycle: 10%, pulse repetition frequency: 1 Hz, and time per spot: 30 s) for 5 min. In the control, saline-injected mice without and with HIFU treatment did not show any therapeutic efficacy during the experiment. However, free DOX, DOX (+HIFU), DOX-CNPs showed a mild inhibitory effect on tumor growth, indicating free DOX was not enough to kill A549 tumor cells. Moreover, DOX-CNPs did not present an enhanced therapeutic efficacy of drug-loaded nanoparticles in ECM-rich tumor tissues due to the limited deep tumor penetration effect. In particular, HIFU-treated DOX-CNPs showed an improved therapeutic efficacy, compared to free DOX and DOX-CNPs without HIFU treatment. At 22 days post-treatment, the mean tumor volumes of DOX-CNPs (+HIFU) were greatly suppressed to 110.46 ± 18.52 mm^3^, compared to free DOX (+HIFU) (197.01 ± 21.22 mm^3^) and DOX-CNPs without HIFU treatment (187.77 ± 18.30 mm^3^) (Figure 5a). To demonstrate the organ toxicity of DOX-CNPs after HIFU treatment, H&E-stained tissue images of liver and kidney after 22 days post-injection confirmed that there was no organ toxicity (Figure S5). Furthermore, all animal groups showed no changes in survival rate during the treatment (Figure 5b). These results indicate that the HIFU treatment of DOX-CNPs could greatly increase the antitumor efficacy in ECM-rich tumor models, resulting in the deep tumor penetration of drug-loaded nanoparticles at targeted tumor tissues.

## 4. Conclusions

We studied the drug delivery efficacy and therapeutic efficacy of HIFU-triggered drug-loaded nanoparticles on ECM-rich tumor models. Doxorubicin-loaded glycol chitosan nanoparticles (DOX-CNPs) could be well accumulated in ECM-rich tumor tissues through HIFU treatment. The in vitro HIFU-triggered DOX-CNPs showed enhanced cellular uptake and rapid drug release from drug-loaded nanoparticles in cultured cells, resulting in increased cytotoxicity, compared to DOX-CNPs without HIFU treatment. In addition, when ECM-rich A549 tumor tissues were treated with HIFU, DOX-CNP could be effectively accumulated at targeted tumor tissues via deep tumor penetration, wherein the dense ECM is broken by external HIFU. Based on these results, HIFU treatment on ECM-rich tumor tissues could increase the accumulation efficiency of DOX-CNPs and inhibit efficiently tumor growth. Therefore, the improved antitumor efficacy of HIFU-triggered DOX-CNPs can be successfully applied to heterogeneous cancer treatments.

## Supplementary Materials

The following are available online at https://www.mdpi.com/1999-4923/12/10/974/s1, Figure S1: TEM size histogram and volume-weighted size distribution of CNPs and DOX-CNPs, Figure S2: Quantitative analysis of cellular uptake of free DOX and Cy5.5-labeled DOX-CNPs in A549 cancer cells, Figure S3: Collagen matrix in SCC7 and A549 tumor tissues that were stained with Masson’s trichrome staining, Figure S4: Masson’s trichrome staining images of A549 tumor tissues without or without HIFU treatment, Figure S5: Liver and kidney H&E staining images to demonstrate organ toxicity of DOX-CNPs after HIFU treatment.
