# Supplementary material for: Deep Tumor Penetration of Doxorubicin-Loaded Glycol Chitosan Nanoparticles Using High-Intensity Focused Ultrasound
Source: Pharmaceutics. 2020 Oct 15;12(10):974. doi: 10.3390/pharmaceutics12100974 (PMC7650702; doi:10.3390/pharmaceutics12100974)
Supplement: Supplementary file 1 [file pharmaceutics-12-00974-s001.zip › pharmaceutics-943159-supplementary.docx]

Supplementary Materials: Deep Tumor Penetration of Doxorubicin-Loaded Glycol Chitosan Nanoparticles using High-Intensity Focused Ultrasound

Yongwhan Choi, Hyounkoo Han, Sangmin Jeon, Hong Yeol Yoon, Hyuncheol Kim, Ick Chan Kwon * and Kwangmeyung Kim *


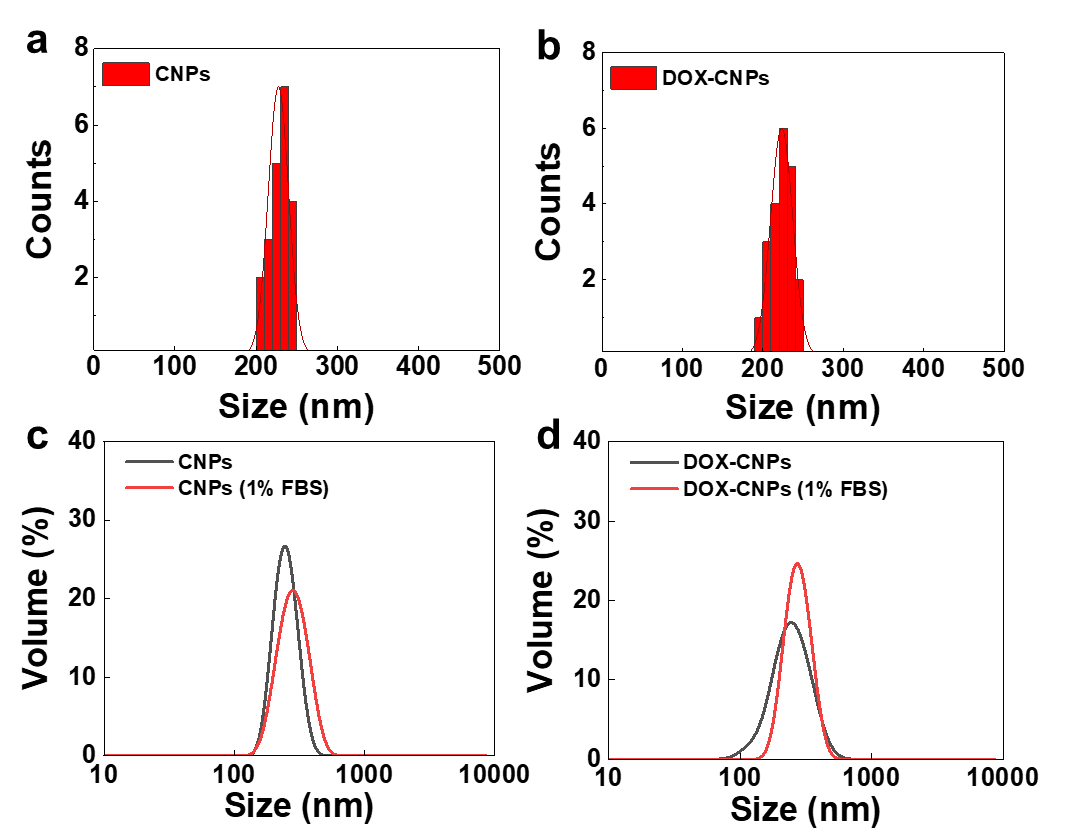


**Figure S1.** The size distribution of (**a**) CNPs and (**b**) DOX-CNPs measured using TEM images (*n* = 10). (**c**) The volume-weighted size distribution of CNPs dispersed in PBS (pH 7.4) and 1% FBS-containing PBS (pH 7.4). (**d**) The volume-weighted size distribution of DOX-CNPs dispersed in PBS (pH 7.4) and 1% FBS contained PBS (pH 7.4). The volume-weighted size distribution was measured using DLS (Nano ZS, Malvern Panalytical Ltd., Grovewood Road, UK) at 25 °C.

**Figure S2.** Quantitative analysis of cellular uptake of free DOX and Cy5.5-labeled DOX-CNPs in A549 cancer cells. A549 cancer cells were incubated with free DOX (1 μg/mL) and DOX-CNPs (10 μg/mL) without or with pre-treatment of US exposure for 10 and 30 min, respectively. DOX-CNP-treated A549 cells were pre-treated in US destruction mode (power: 10 MHz, mechanical index: 0.235) for 5 min. The fluorescence intensity of free DOX and Cy5.5-labeled DOX-CNPs was quantified using the Image-Pro Plus software.


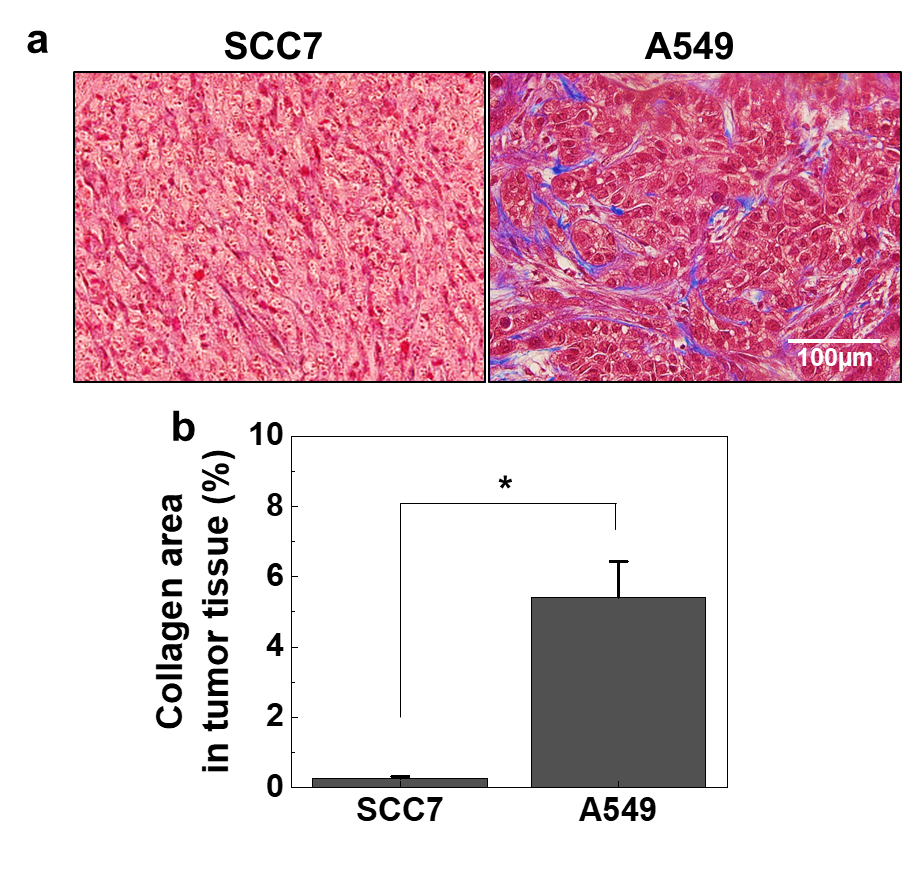


**Figure S3.** (**a**) Collagen matrix in SCC7 and A549 tumor tissues that were stained with Masson's trichrome staining (blue color). (**b**) Quantitative analysis of collagen fibers in SCC7 and A549 tumor tissues. Collagen contents = blue stained area/total area x 100 (%). (*) indicates difference at the *p* < 0.05 significance level.


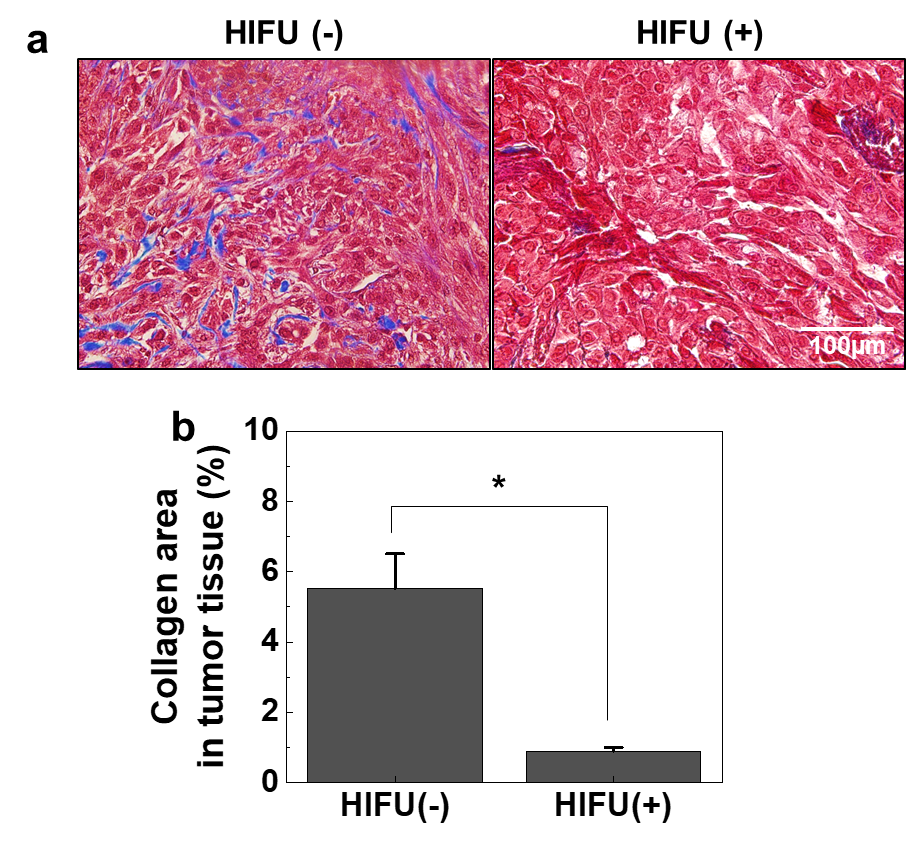


**Figure S4.** Masson's trichrome staining images of A549 tumor tissues without or without HIFU treatment. (**a**) After 6 h post-treatment of HIFU for 5 min, tumor tissues were excised and the tissue slides were stained with Masson’s trichrome staining. (**b**) Quantitative analysis of collagen fibers in A549 tumor tissues. Collagen area = blue stained area/total area x 100 (%). (*) indicates difference at the *p* < 0.05 significance level.


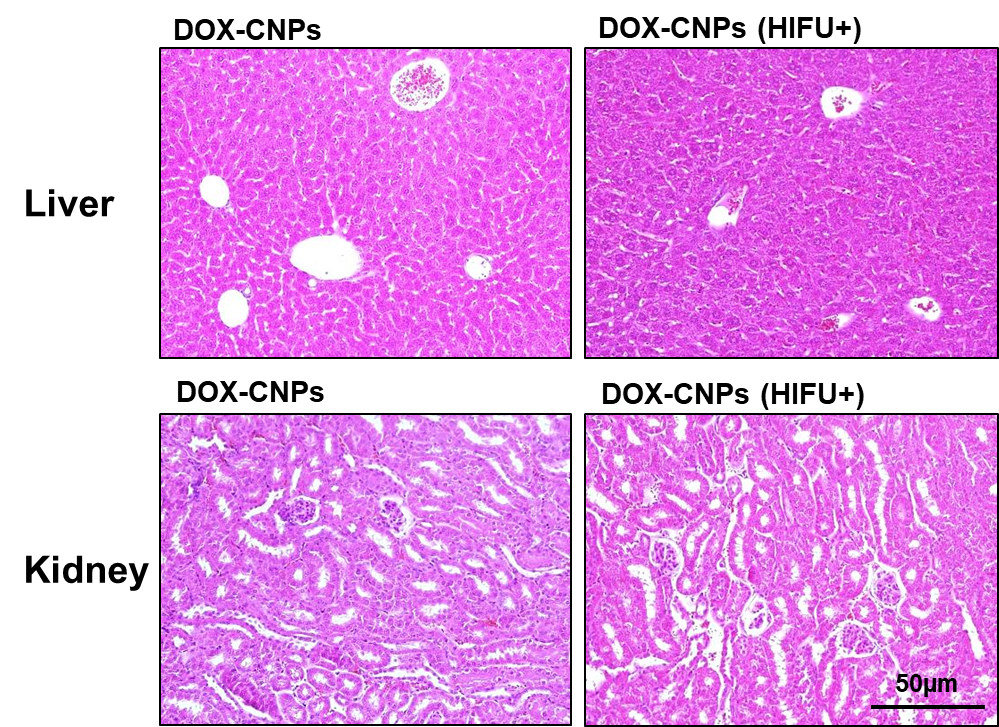


**Figure S5.** H&E staining images of liver and kidney to demonstrate non-toxicity of DOX-CNPs without and with HIFU treatment after 22 day post-treatment.
